# The Association of Receiving a Diagnosis of Idiopathic Scoliosis with the Self-Esteem and Mental Health of Pediatric Patients: A Cohort Study

**DOI:** 10.3390/children13070946

**Published:** 2026-07-18

**Authors:** Petar Kaliterna, Ivan Buljan, Ivana Šegvić, Mislav Čimić, Marija Franka Žuljević

**Affiliations:** 1 Department of Physical Medicine and Rehabilitation, Polyclinic for the Rehabilitation of Persons with Developmental Disabilities, 21000 Split, Croatia; ivanasegvic@hotmail.com; 2 Department of Psychology, Faculty of Humanities and Social Sciences, University of Split, 21000 Split, Croatia; ibuljan@ffst.hr; 3 Clinical Hospital Centre Zagreb, Clinical Department of Orthopaedic Surgery, School of Medicine, University of Zagreb, 10000 Zagreb, Croatia; cimicmislav@gmail.com; 4 Centre for Evidence-Based Medicine, School of Medicine, University of Split, 21000 Split, Croatia; marija.franka.zuljevic@mefst.hr

**Keywords:** adolescent idiopathic scoliosis, self-esteem, mental health

## Abstract

**Highlights:**

**What are the main findings?**

- Adolescent idiopathic scoliosis diagnosis was associated with an immediate drop in the self-esteem and mental health of patients.
- Partial emotional adaptation is present, even though residual depressive symptoms may persist in adolescents with more severe curves.

**What are the implications of the main findings?**

- Assessment for mental health issues after receiving a scoliosis diagnosis should be the standard of care.
- Interdisciplinary team care models should be standard for scoliosis patients, especially those with severe curves.

**Abstract:**

Background/Objectives: Patients with adolescent idiopathic scoliosis (AIS) are at increased risk of emotional distress, yet little is known about the immediate psychological impact of receiving the diagnosis itself. This study aimed to evaluate short-term changes in self-esteem, anxiety, depression, and stress before and after AIS diagnosis, and in the early post-diagnostic period. Methods: In this cohort study, 179 adolescents (10–18 years) referred for suspected AIS completed the Rosenberg self-esteem scale (RSES) and Depression, Anxiety and Stress Scale (DASS 21) before clinical and radiographic assessment (T1). Based on clinical findings and Cobb angle measurements, participants were classified as having mild scoliosis (Cobb angle > 10°, <20°) managed with observation alone (Group A, *n* = 30), moderate/severe scoliosis (Cobb angle > 20°), candidates for bracing treatment (Group B, *n* = 30), or no scoliosis (Group C, *n* = 119). Adolescents with scoliosis (Groups A and B) repeated the questionnaires immediately after diagnosis and treatment recommendations (T2). Group B completed a third assessment 1–2 months after initial examination (T3). Results: At baseline, RSES and DASS 21 scores did not differ significantly between groups. Following diagnostic disclosure, Group A showed a significant decline in self-esteem, while both groups showed increases in stress, anxiety, and depression. In Group B, reassessed at T3, stress and anxiety decreased, whereas depression remained elevated relative to baseline. Conclusions: AIS diagnosis was associated with a self-esteem decline that followed a pattern dependent on curve severity, with an immediate drop in mild AIS (Group A) versus a delayed decline emerging one to two months later in moderate/severe cases (Group B). Anxiety and depressive symptoms increased significantly in both groups, while stress increased significantly only in those with moderate/severe scoliosis (Group B). Routine screening of mental health after AIS diagnosis, alongside interdisciplinary care involving mental health professionals, may help identify vulnerable patients and enable timely psychosocial support.

## 1. Introduction

Scoliosis, a three-dimensional deformity of the spine, alters normal body morphology [1]. Adolescent idiopathic scoliosis (AIS) is the most common type, with an estimated prevalence of approximately 0.5–5% (often quoted as 2–4%) among adolescents and a female-to-male ratio ranging from about 1.5:1 to 3:1, increasing with age and curve severity [1,2,3]. These years are characterized not only by rapid physical growth but also by significant psychological development, making adolescents particularly sensitive to body-related perceptions [4]. Adolescents with visible physical differences often report reduced body satisfaction and self-esteem, partly due to increased self-consciousness and social comparison [4,5]. Among patients with AIS, movement limitations and the need for visible orthopedic bracing can intensify feelings of stigma, frustration, and negative body image, thereby further reducing mental health [5,6,7].

Self-esteem (SE) refers to an individual’s overall evaluation of their own worth and plays a fundamental role in shaping how people view and relate to themselves [8]. Fluctuations in SE are associated with emotional instability, aggression, and personality traits linked to the experience of positive or negative affect [9]. Low SE during childhood has been shown to negatively influence emotional regulation, increase the risk of depression, and reduce satisfaction with relationships, career achievements, and physical health [10]. Moreover, persistent low SE in adolescence and early adulthood is associated with poorer life outcomes, including criminal behavior and limited economic opportunities [11]. It also contributes to body dissatisfaction during adolescence, a period when self-image is especially vulnerable [12,13].

Numerous studies have evaluated the psychological and emotional impact of scoliosis and its treatments on adolescents. Studies suggest that AIS is frequently associated with lower quality of life, emotional distress, and increased risk of depression, regardless of treatment type, whether observation, bracing, or surgery [4,5,6,7,14,15]. Sanders et al. [16] noted that clinically significant psychological disturbances can emerge in AIS patients undergoing various forms of management. However, findings are not uniformly negative. Goldberg et al. [17] reported that many adolescents with scoliosis developed adaptive coping mechanisms and maintained a reasonably positive self-perception despite poorer perceived body image and physical limitations, particularly among females.

Despite this evidence, little is known about how the moment of receiving an AIS diagnosis influences adolescents’ psychological well-being, including self-esteem, anxiety, depression, and stress levels. Understanding this initial psychological response is crucial, as it may guide timely interventions and psychological support for affected patients and help clinicians identify those at risk of poorer long-term adjustment [18]. Moreover, most existing research has focused on the broader, long-term psychosocial impact of scoliosis and its treatment [4], rather than on the immediate pre- and post-diagnostic period when perceptions of illness, body image, and future expectations are first being shaped. This early period may represent a distinct window of psychological vulnerability, as adolescents must process the diagnosis before treatment-related coping mechanisms are in place, underscoring the need for targeted evidence on this specific timeframe. Therefore, the aim of this study was to evaluate changes in self-esteem, anxiety, depression, and stress among patients before and after receiving a diagnosis of scoliosis, as well as during the early post-diagnostic period, using validated psychological assessment questionnaires. We hypothesized that receiving a diagnosis of scoliosis would be associated with a measurable decline in self-esteem and an increase in anxiety, depression, and stress levels, with these psychological changes persisting, at least in part, into the early post-diagnostic phase.

## 2. Materials and Methods

### 2.1. Study Design and Setting

This was a cohort study. The research was conducted between January 2025 and July 2025 by a team based in Split, Croatia. Patient selection and data collection were carried out at the Polyclinic for the Rehabilitation of Persons with Developmental Disabilities in Split. The Polyclinic is the main scoliosis screening center for adolescents in Split.

### 2.2. Participants

Our study included adolescents aged 10 to 18 years who came to the Polyclinic for an initial examination due to suspected adolescent idiopathic scoliosis (AIS). Adolescents were eligible if this was their first examination for suspected AIS and they had no prior diagnosis. We excluded those with other structural spinal diseases such as kyphosis or congenital spinal defects, those with a diagnosed mental illness, and individuals younger than 10 or older than 18 years. All patients who were eligible according to the criteria were included.

Scoliosis status was determined with clinical features (shoulder and scapula asymmetry, waist or trunk shift asymmetry) and Adam’s forward bend test (rib hump or lumbar prominence on forward bending) [19] and confirmed radiographically using standard standing spine radiographs, with the Cobb angle measured as the gold standard for curve magnitude. A diagnosis of adolescent idiopathic scoliosis was confirmed when the Cobb angle was greater than 10°, consistent with established criteria [20].

Following clinical and radiographic evaluation, the participants were categorized into three groups based on clinical and radiological findings. Group A consisted of adolescents who met the clinical criteria for scoliosis and had a Cobb angle greater than 10° but less than 20° on radiographs, corresponding to mild scoliosis managed with observation alone. Group B included adolescents with clinical signs of scoliosis and a Cobb angle of 20° or greater on radiographs, corresponding to at least moderate scoliosis meeting the threshold for brace treatment. Group C (the control group) included adolescents who did not meet the clinical criteria for scoliosis on physical examination and therefore did not have radiographic evidence of scoliosis.

### 2.3. Variables and Data Collection

The primary outcome measure in the quantitative part of this study was the Rosenberg self-esteem scale (RSES). The RSES was developed to describe, evaluate, and predict self-esteem, as well as to monitor changes resulting from therapeutic interventions [21]. It is among the most widely used, valid, and reliable measures of SE [22]. The scale consists of 10 items assessing both positive and negative self-perceptions to measure global self-esteem. Each item is rated on a 4-point Likert scale ranging from strongly disagree to strongly agree. Items 2, 5, 6, 8, and 9 are reverse-scored. The total score reflects the participant’s overall level of self-esteem, with higher scores indicating greater self-esteem. The internal consistency (Cronbach’s alpha) of RSES was 0.76 (95% CI, 0.71 to 0.81).

Along with the RSES, the participants completed the Depression, Anxiety, and Stress Scale (DASS-21), a validated instrument widely used to assess symptoms of depression, anxiety, and stress [23,24]. Each item is rated on a 4-point Likert scale ranging from 0 (“Did not apply to me at all”) to 3 (“Applied to me very much”). Higher scores on each subscale indicate greater severity of the respective symptomatology. The internal consistency of DASS-21 was α = 0.88 (95% CI, 0.87 to 0.91), and for each subsection: Depression α = 0.72 (95%CI, 0.61 to 0.83), Anxiety α = 0.77 (95% CI, 0.70 to 0.83), and Stress α = 0.75 (95% CI, 0.67 to 0.83).

Before the clinical examination, all the participants completed pen-and-paper versions of the RSES and the DASS-21, representing the baseline assessment (T1). Age was recorded as a continuous variable, and sex was included as a categorical variable in descriptive analyses. Participants with scoliosis (Groups A and B) completed the same questionnaires again immediately after receiving the diagnosis, which was confirmed with X-rays on the same day as the initial exam, to assess short-term post-diagnosis changes in self-esteem, depression, anxiety, and stress (T2). Participants in Group B (moderate or severe scoliosis) were prescribed brace treatment using a Chêneau brace in accordance with current conservative management principles for AIS. These participants then returned for a follow-up examination approximately one to two months after the initial visit, once they had received and begun wearing the brace, and again completed the RSES and DASS-21 to evaluate changes in the examined psychological domains over time and after initiation of bracing (T3).

### 2.4. Study Size

We calculated the sample size based on the study by Zhang et al. [5], which compared the treated and nontreated groups for AIS, using the RSES. The untreated group had M = 24 (Sd = 3), and the treated group had M = 28 (Sd = 4). With an alpha level of 0.05 and 80% power, we calculated that we would need at least 28 total (14 per group).

From the same study, we calculated the sample size for within-group comparison. Based on the parameters related to self-esteem levels in the surgical group before treatment, M = 24 (Sd = 3), and after treatment (M = 30, Sd = 2), we calculated that only 6 participants were necessary to determine the effect of that size. However, we decided to collect a minimum of double the sample, due to potential attrition. The calculation was made by using G Power version 3.1.

### 2.5. Statistical Methods

Continuous variables were assessed for distribution and summarized using medians (M) and interquartile ranges (IQR), while categorical variables were described using frequencies and percentages. Baseline differences between the three study groups (mild scoliosis, moderate/severe scoliosis, and controls) were evaluated using the Kruskal–Wallis test for continuous variables and the χ^2^ test for categorical variables.

Changes in psychological outcomes over time were analyzed using non-parametric tests due to the non-normal distribution of questionnaire scores. For participants with mild scoliosis (Group A), differences between baseline (T1) and post-diagnosis (T2) measurements were assessed using the Wilcoxon signed-rank test. For participants with moderate/severe scoliosis (Group B), repeated measurements across three time points, T1, T2, and T3, were analyzed using the Friedman test, followed by Conover post hoc pairwise comparisons when significance was detected. Rank-biserial correlation (r) with 95% confidence interval (CI) and Kendall’s W (W) were used as effect size measures for within-group changes.

All statistical tests were two-tailed, and a *p* value < 0.05 was considered statistically significant. Statistical analyses were performed using R statistical software (version R-4.5.3, R Foundation for Statistical Computing, Vienna, Austria) and jamovi (version 2.6.17, the jamovi project, Sydney, Australia).

### 2.6. Ethical Considerations

Written informed consent was obtained from the parents, and written informed assent was obtained from all participants. The study was conducted in accordance with the Declaration of Helsinki, and approved by the Ethics Committee of the School of Medicine, University of Split (protocol code 2181-198-03-04-24-0116, 23 December 2024). This study was conducted and reported in accordance with the STROBE (Strengthening the Reporting of Observational Studies in Epidemiology) statement [25].

## 3. Results

Overall, 179 participants (69 male and 110 female), who were eligible according to the inclusion and exclusion criteria, were enrolled in the study after completing the RSES and DASS-21 questionnaires prior to the initial clinical examination (T1). The participants were subsequently classified into three groups based on clinical and radiological assessment: Group A—mild scoliosis (*n* = 30); Group B—moderate/severe scoliosis (*n* = 30); and Group C—no scoliosis (*n* =119). The median age of the sample was 12 years (interquartile range 11–14). There was a significantly higher prevalence of female participants in Group B (83.3%), compared to Groups A (63.3%) or C (55.5%). Likewise, participants from Group B had a higher median age than participants in Group C. Age differed significantly between groups (Kruskal–Wallis *p* < 0.001), with post hoc comparisons indicating a higher median age in Group B compared with Group C (Table 1).

**Figure 1:**
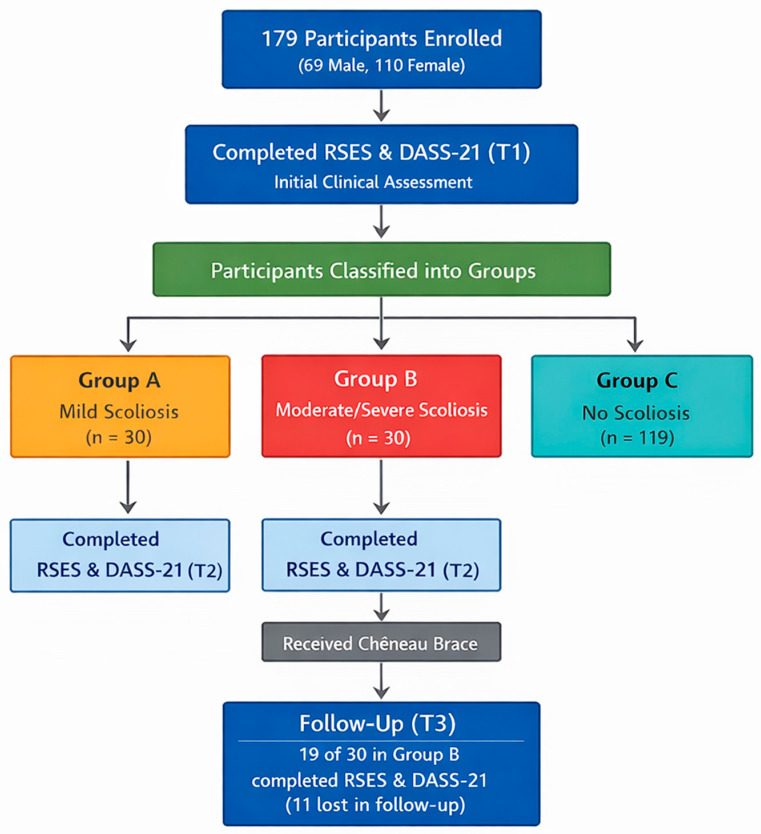
Flow chart of participant enrolment.

Following confirmation of the scoliosis diagnosis, the participants in Groups A (*n* = 30) and B (*n* = 30) again completed the RSES and DASS-21 at T2. All questionnaires were immediately checked for completeness, resulting in no missing data at T1 or T2. After approximately one to two months (minimum 31 days, maximum 61 days since follow-up), patients in Group B had received a Chêneau brace and were scheduled for a follow-up visit, during which they completed the questionnaires for the third time (T3). Attrition at this stage was substantial, with only 19 of the 30 patients in Group B attending the follow-up in the planned assessment window (Figure 1).

### 3.1. Baseline Self-Esteem, Anxiety, Stress, and Depression Scores

There were no statistically significant baseline differences in psychological measures between the three groups. At T1, median Rosenberg self-esteem scale scores were similar between the mild, severe, and control groups (36, 36, and 35, respectively; *p* = 0.069), indicating comparable baseline self-esteem. DASS-21 stress, anxiety, and depression scores were low at baseline in all groups, with no statistically significant between-group differences (all *p* > 0.05), suggesting similar initial emotional functioning regardless of scoliosis status (Table 2).

### 3.2. Changes After Diagnosis (T1–T2)

Following clinical and radiological confirmation of scoliosis (T2), a decline in self-esteem was observed in both scoliosis groups: Median RSES scores decreased from 36 to 33 in Group A (r = 0.36, 95% CI 0.08 to 0.59), *p* < 0.05. In Group B, a numerical decrease from 36 to 34 was observed but did not reach statistical significance. Over the same interval, DASS-21 indices showed a consistent worsening of emotional symptoms. In Group B, stress increased from 5 to 8 (r = 0.48, 95% CI 0.23 to 0.68), anxiety from 2 to 4 (r = 0.50, 95% CI 0.25 to 0.69), and depression from 1 to 3 (r = 0.41, 95% CI 0.14 to 0.62), all representing statistically significant deteriorations from T1 (*p* < 0.01). In Group A, anxiety increased from 1 to 2 (r = 0.30, 95% CI 0.01 to 0.54) and depression from 0 to 2 (r = 0.43, 95% CI 0.16 to 0.43), both statistically significant (*p* < 0.01), while stress showed a numerical increase from 5 to 8 that did not reach statistical significance (Table 2). Notably, despite these statistically significant increases, DASS-21 scores remained within normal severity ranges throughout, with the worst-affected subgroup (Group B, T2) still classified as normal for depression and only reaching the mild threshold for anxiety and stress according to standard severity banding.

### 3.3. Changes After Follow-Up (T3)

**Figure 2:**
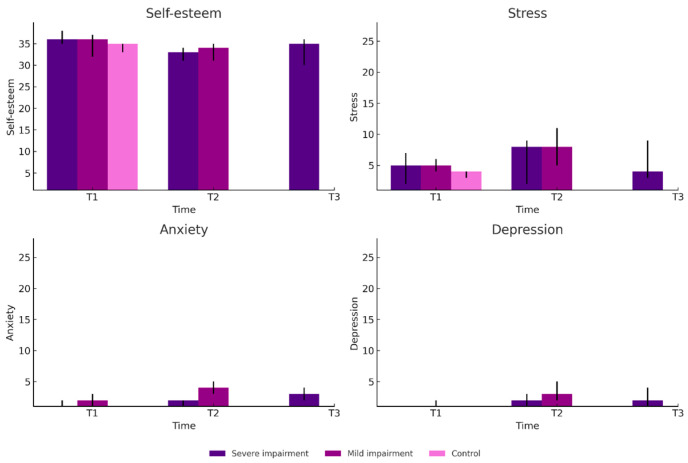
Visual representation of results: median levels of self-esteem, stress, anxiety and depression in three groups (severe scoliosis, mild scoliosis, no scoliosis) across three time points before first examination (T1), after (T2) and at the follow-up examination (T3).

At T3, assessed approximately one to two months after brace prescription, data were collected only for adolescents with moderate/severe scoliosis (Group B). In this group, median RSES scores showed a numerical increase from 34 at T2 to 35 at T3, though this change was not statistically significant. Self-esteem at T3 remained significantly lower than baseline (T1) (W = 0.16, *p* < 0.05), indicating incomplete recovery. DASS-21 stress scores decreased from 8 to 4 (W = 0.43), and anxiety scores declined from 4 to 3 (W = 0.38), with anxiety showing significant improvement compared with both T1 and T2, whereas depression scores remained at a median of 2 (W = 0.27), still elevated relative to baseline despite some numerical reduction (*p* < 0.01) (Table 2). A visual representation is available in Figure 2.

## 4. Discussion

At baseline, adolescents with mild AIS, moderate/severe AIS, and no scoliosis had comparable self-esteem and low DASS 21 scores, suggesting that referral for suspected scoliosis alone may not be associated with impaired mental health. The gender distribution is consistent with findings in the scoliosis literature, where females are more frequently affected [2]. Patterns over time in our results suggest that receiving a scoliosis diagnosis is associated with short-term psychological changes that varied by domain and group: self-esteem declined significantly in patients with mild scoliosis, while anxiety and depressive symptoms increased significantly in both groups, and stress increased significantly only in patients with moderate/severe scoliosis. These changes were followed by partial emotional adaptation over the subsequent one to two months, although residual depressive symptoms may persist in adolescents with more severe curves. Although statistically significant, the observed decline in RSES scores in patients with mild scoliosis (3 points) falls at or below the commonly cited minimally clinically important difference of 3–5 points. The numerical decline in patients with moderate/severe scoliosis (2 points) did not reach statistical significance. This suggests that while the acute psychological response is measurable, its clinical relevance may be modest for many patients.

Notably, self-esteem appeared to follow a different temporal trajectory depending on curve severity. Patients with mild scoliosis (Group A) showed an immediate, statistically significant decline in self-esteem at the time of diagnosis (T2), whereas patients with moderate/severe scoliosis (Group B) showed no significant acute change at T2; their self-esteem only became significantly lower than baseline at T3, one to two months later. This suggests that mild and moderate/severe AIS may be associated with distinct patterns of self-esteem response: an immediate drop in the case of mild curves, versus a delayed decline in the case of more severe curves, a distinction that has not been consistently drawn in the existing literature [4,12], which has largely treated self-esteem change as a uniform finding across severity groups. Similarly, although changes in DASS-21 scores reached statistical significance, they remained largely subclinical in magnitude: with standard severity banding, depression stayed within the normal range, while anxiety and stress reached only the mild threshold.

The baseline scores align with the literature indicating that not all adolescents with AIS have clinically relevant distress at first presentation, although AIS overall is a risk factor for mental health problems [4,16]. Changes in mental health parameters are consistent with reviews and cohort studies reporting elevated anxiety, depressive symptoms, and psychological distress among AIS patients across treatment stages [12,26]. However, several studies indicate that many adolescents with AIS do not experience a clear short-term spike in internalizing symptoms. For example, a recent longitudinal cohort found that adolescents with AIS managed differently (observation, bracing, or surgery) showed no significant change in emotional distress scores over two years, suggesting relative stability rather than an early post-diagnosis worsening [27]. A 2025 quality of life study found that only about one-third of adolescents with scoliosis had reduced psychological or physical well-being, while most scored within the normal range [28]. In the context of self-esteem, D’Agata et al. [29] reported that over six months, adolescents with higher baseline self-esteem improved in body satisfaction, while those with lower baseline self-esteem improved in self-image, suggesting that many patients show stable or improving self-related perceptions rather than early deterioration. This pattern complements our findings of partial adaptation and gradual strengthening of self-related perceptions.

Our study was limited by a potential source of selection bias, as we only included patients who were willing to complete questionnaires before their first examination. This approach may have excluded individuals who were less motivated, had limited literacy, or faced language barriers, leading to a non-representative sample. Also, participant dropout in the final stage (T3) was substantial; 11 of 30 patients in Group B did not attend the follow-up exam, potentially due to apprehension regarding brace treatment, delays in brace provision leading to missed assessment windows, or other factors such as intercurrent illness or failure to attend the appointment. However, the exact reasons for this loss to follow-up remain unknown, and since we did not perform a formal comparison between completers and those lost to follow-up, we cannot rule out the possibility of attrition bias. Another limitation of this study is that Group C was not reassessed at the second time point (T2). As a result, the observed changes from T1 to T2 in the other groups cannot be directly compared against a corresponding change in a control group, limiting our ability to distinguish the psychological effect of diagnostic disclosure from other factors, such as the clinical consultation itself or the effect of repeated questionnaire administration. The study was conducted in a single regional center in Croatia, which may limit external generalizability to other health systems and cultural settings. The short follow-up period (1–2 months) captures only early adaptation, and psychological trajectories during several years of brace treatment or after surgery may differ. Finally, potential confounders such as socioeconomic status, family functioning, and social support were not measured, so residual confounding and effect modification cannot be ruled out. Additionally, baseline differences in age and sex between groups were not adjusted for in the analyses, and given their known influence on self-esteem and mental health outcomes, these variables should be considered potential confounders. Furthermore, our study was not originally designed as a longitudinal study, which constrained our statistical options. A longitudinal design, paired with a mixed-effects approach, would allow for more robust handling of repeated measurements and potential confounders. Future research with larger samples and longitudinal designs should examine whether a confounding effect exists.

Clinically, these findings suggest that clinicians may consider systematic assessment of mental health at and shortly after AIS diagnosis, not only during later treatment, because a substantial proportion of patients show a slight acute decline in self-esteem and emotional state at this early stage. Brief validated tools such as the DASS 21 and simple self-esteem or quality of life scales could be integrated into routine visits to identify high-risk patients and guide early psychosocial interventions, such as brace compliance monitoring with counseling, coping skills, and psychoeducational programs, structured nurse-led psychological care, and family-involved mental health support after surgery [30,31]. Given the evidence that brace treatment can negatively affect quality of life and body image [7], interdisciplinary care models including orthopedic teams, physiotherapists, and mental health professionals should be considered the standard of care for moderate/severe AIS [32].

Future research should include multicenter cohorts with longer follow-up to describe trajectories of self-esteem, anxiety, depression, and stress from diagnosis through the full course of brace treatment and potential surgery. Studies that incorporate detailed psychosocial variables like body image, perceived stigma, family support, and coping strategies would help clarify causal pathways and identify modifiable targets. Finally, controlled trials of structured psychological interventions like cognitive behavioral or digital support programs [33] initiated at diagnosis or brace prescription are needed to determine whether early, targeted support can prevent or mitigate the acute decline in mental health observed in AIS patients.

## 5. Conclusions

In this cohort of adolescents referred for suspected AIS, baseline self-esteem and mental health status were similar in those with and without scoliosis, suggesting that referral alone does not necessarily imply impaired mental health. However, the moment of diagnostic disclosure was associated with measurable short-term psychological changes: a significant decline in self-esteem in patients with mild scoliosis, and significant increases in anxiety and depressive symptoms in patients with both mild and moderate/severe scoliosis, while a significant increase in stress was observed only in patients with moderate/severe scoliosis.

Among adolescents with more pronounced curves, partial recovery of self-esteem and improvement in stress and anxiety were observed within 1–2 months. Still, depressive symptoms remained higher than at baseline, indicating incomplete psychological adaptation in the early post-diagnostic period. These patterns highlight the potential importance of systematic, early mental health assessment after AIS diagnosis, using brief validated tools such as the DASS-21 and self-esteem measures to detect those at greater risk. Integrating orthopedic care with physiotherapy and structured psychological or family-based support should be considered standard practice, particularly for adolescents prescribed bracing, to mitigate the acute decline in mental health status associated with learning about AIS and starting treatment.

## Figures and Tables

**Table 1:** Demographic data of study participants (*N* = 179).

		Group A—Mild Scoliosis (*n* = 30)	Group B—Moderate/Severe Scoliosis (*n* = 30)	Group C—No Scoliosis (*n* = 119)	Total (*N* = 179)	*p*
Age (M, IQR)		12 (11–15)	13.5 (12–14.8)	12 (11–14)	12 (11–14)	<0.001 *
Gender (*N*, %)						
	Male	11 (36.7)	5 (16.7)	53 (44.5)	69 (38.5)	0.019 **
	Female	19 (63.3)	25 (83.3)	66 (55.5)	110 (61.5)	

Abbreviations: M = median, IQR = interquartile range. * Kruskal–Wallis test. Dwass–Steel–Critchlow–Fligner pairwise comparison showed that only Groups B and C differ (*p* < 0.001). ** Chi-square test.

**Table 2:** Rosenberg self-esteem scale (RSES) and Depression, Anxiety and Stress Scale (DASS-21) scores over all measurement points (*N* = 179).

	Variables	Group A—Mild Scoliosis (*n* = 30)	Group B—Moderate/Severe Scoliosis (*n* = 30)	Group C—No Scoliosis (*n* = 119)	*p* *
**Measurement points**		**Median (IQR)**			
**T1—prior to the initial clinical examination**	Rosenberg self-esteem (RSES)	36 (35–38)	36 (32–37)	35 (33–35)	**0.069**
	DASS-21 Stress	5 (2–7)	5 (4–6)	4 (3–4)	**0.391**
	DASS-21 Anxiety	1 (1–2)	2 (1–3)	1 (1–1)	**0.249**
	DASS-21 Depression	0 (0–1)	1 (0–2)	0 (0–1)	**0.415**
**T2—immediately after receiving the diagnosis**	Rosenberg self-esteem (RSES)	33 (31–34) †	34 (31–35)		
	DASS-21 Stress	8 (2–9)	8 (5–11) †		
	DASS-21 Anxiety	2 (1–2) †	4 (3–5) †		
	DASS-21 Depression	2 (1–3) †	3 (2–5) †		
**T3—follow-up examination**	Rosenberg self-esteem (RSES)		35 (30–36) †		
	DASS-21 Stress		4 (3–9) ‡		
	DASS-21 Anxiety		3 (2–4) †,‡		
	DASS-21 Depression		2 (0–4) †		

Abbreviations: IQR = interquartile range. * Kruskal–Wallis test. † Statistically significant from T1, Wilcoxon test for Group A or Friedman test with Conover post hoc test for Group B. All comparisons yielded *p* < 0.01, except for Rosenberg self-esteem scores (*p* = 0.04). ‡ Statistically significant from T2, Friedman test with Conover post hoc test for Group B. All comparisons yielded *p* < 0.01.

## Data Availability

The original contributions presented in this study are included in the article. Further inquiries can be directed to the corresponding author.

## Abbreviations

The following abbreviations are used in this manuscript:

SE	Self-esteem
AIS	Adolescent idiopathic scoliosis
RSES	Rosenberg self-esteem scale
DASS-21	Depression, Anxiety and Stress Scale
